# Prevalence and genotyping of *Toxoplasma gondii* in questing *Ixodes ricinus* ticks from forest areas of Northern Poland

**DOI:** 10.1007/s10493-024-00965-w

**Published:** 2024-10-09

**Authors:** Małgorzata Adamska

**Affiliations:** Department of Genetics and Genomics, Institute of Biology, University of Szczecin, Felczaka 3c, Szczecin, 71-412 Poland

**Keywords:** Questing *Ixodes ricinus*, *Toxoplasma gondii*, Prevalence, Transmission, Coinfection, Genotyping

## Abstract

*Toxoplasma gondii* occurs in a wide range of intermediate hosts, whose blood may be a meal for different tick species. A few studies have examined the role of ticks in the life cycle of *T. gondii*. This one includes the largest number and all stages of *Ixodes ricinus* collected from the widest area, covering seven recreational localities within a forest biotope in Northern Poland. This study aimed to determine the prevalence of *T. gondii* DNA in 2144 collected questing ticks to establish whether they may be involved in *T. gondii* life cycle. The additional goal was to genotype the detected *T. gondii*, as knowledge about its genotypes occurring in European ticks is insufficient. A further purpose was to detect coinfection with *T. gondii* and *Borreliaceae* in the collected ticks, as all of them have previously been tested for the presence of bacteria DNA. Nested PCR and sequencing of the obtained B1 gene fragment were conducted. *T. gondii* DNA was detected in 0.9% of all ticks (1.1% of nymphs and 0.7% of larvae). The presence of *T. gondii* in unfed larvae and nymphs may indicate the possibility of its vertical transmission. The prevalence of *T. gondii* DNA in ticks collected from individual sites was focal (0-4.3%) and seems to depend on local climatic conditions. Among all examined ticks, 0.3% were coinfected with *T. gondii* and *Borreliella* spp., vs. 0.6% of specimens with a single *T. gondii* infection. The obtained B1 sequences showed the greatest similarity (99.71–100%) to the sequence representing type III.

## Introduction

*Ixodidae* ticks are vectors for many pathogens, e.g. *Borreliaceae*, *Anaplasmataceae*, and *Rickettsiaceae* bacteria or Apicomplexa protozoa such as *Babesia* spp. (Nowak-Chmura 2013; Wodecka and Kolomiiets 2023). *Toxoplasma gondii* is a worldwide spread Apicomplexan parasite. It was detected in environmental samples and different organisms, including ticks (Ben-Harari 2019; Fernández-Escobar et al. 2022). The presence of *T. gondii* in *I. ricinus* is confirmed (Adamska and Skotarczak 2017; Asman et al. 2015, 2017; Gryczyńska et al. 2024; Kocoń et al. 2020; Sroka et al. 2003, 2008, 2009). The parasite was also detected in other tick species: *Ixodes amblyomma*, *I. turdus*, *Dermacentor reticulatus*, *Haemaphysalis flava*, *H. longicornis*, *Amblyomma cajennense*, and *Rhipicephalus* spp. (Ben-Harari 2019; Ergunay et al. 2022; Kim et al. 2020; Truong et al. 2022; Wójcik-Fatla et al. 2015; Zając et al. 2017).

The life cycle of *T. gondii* is complex. It includes asexual proliferation in a wide range of intermediate hosts such as humans, rodents, or ruminants, and sexual recombination in felines, definitive hosts (Quiarim et al. 2021; Warschkau and Seeber 2023). The human infection is mostly asymptomatic, but severe disease frequently occurs in patients with immunodeficiency or congenital infection. The parasite also causes infections in domestic and wild animals and is associated with economic losses in several livestock species (Ben-Harari 2019; Fernández-Escobar et al. 2022; Quiarim et al. 2021). Transmission of *T. gondii* occurs mainly through consuming raw or undercooked meat containing tissue cysts or the intake of food and water contaminated with sporulated oocysts. Transmission through blood transfusion and organ transplantation is also possible (Ben-Harari 2019; Warschkau and Seeber 2023). Experiments on mice have shown the possibility of *T. gondii* transmission via infected *I. ricinus* during blood sucking (Deryło et al. 1978) or through mice inoculation with tick homogenates (Sroka et al. 2008). Experimental transmission of *T. gondii* by *Dermacentor variabilis*, *D. andersonii*, and *Amblyomma americanum* was also successfully conducted (Woke et al. 1953). In contrast, attempts to demonstrate *T. gondii* transmission by *Haemaphysalis longicornis* (Zhou et al. 2016) and *Ornithodoros moubata* (Jagow and Hoffmann 1970; Ben-Harari 2019) were ineffective.

Genotyping methods have divided *T. gondii* into three clonal lineages: type I, II, and III. At the same time, the genome-wide polymorphism rate between them has been estimated to be approximately 1%. Later studies showed the presence of recombinant variants of these lineages, local or regional clonal lineages, and unique or atypical genotypes. Most European isolates represent one of the three main types (I, II, or III). Type II predominates in Europe, followed by type III. However, type I predominates in European ticks. The genotypes of *T. gondii* have been associated with their pathogenicity; atypical ones and type I are more pathogenic than others (du Plooy et al. 2023; Fernández-Escobar et al. 2022; Quiarim et al. 2021).

In this study, the largest number of *I. ricinus* ticks, collected from the widest area was examined to detect *T. gondii* DNA, compared to other studies on this topic. This study aimed to determine the prevalence of *T. gondii* in all stages of questing *I. ricinus* ticks collected from seven recreational localities within forest areas in northern Poland. Determining the prevalence of *T. gondii* in *I. ricinus* will help establish if they may be involved in the parasite’s life cycle in the studied area. Unlike studies by other authors, this one includes questing larvae. Examination of questing and unfed individuals can help determine whether vertical transmission of *T. gondii* may occur. The next goal was to determine genetic diversity and genotyping of *T. gondii* strains detected in *I. ricinus* ticks, as knowledge about *T. gondii* genotypes occurring in European tick populations is still insufficient. All ticks examined in this study have previously been tested for the presence of DNA of *Borreliaceae* bacteria (Wodecka and Kolomiiets 2023). Another purpose was to detect coinfection of the ticks with bacteria and *T. gondii*.

## Materials and methods

### Ticks collection and identification

**Fig. 1:**
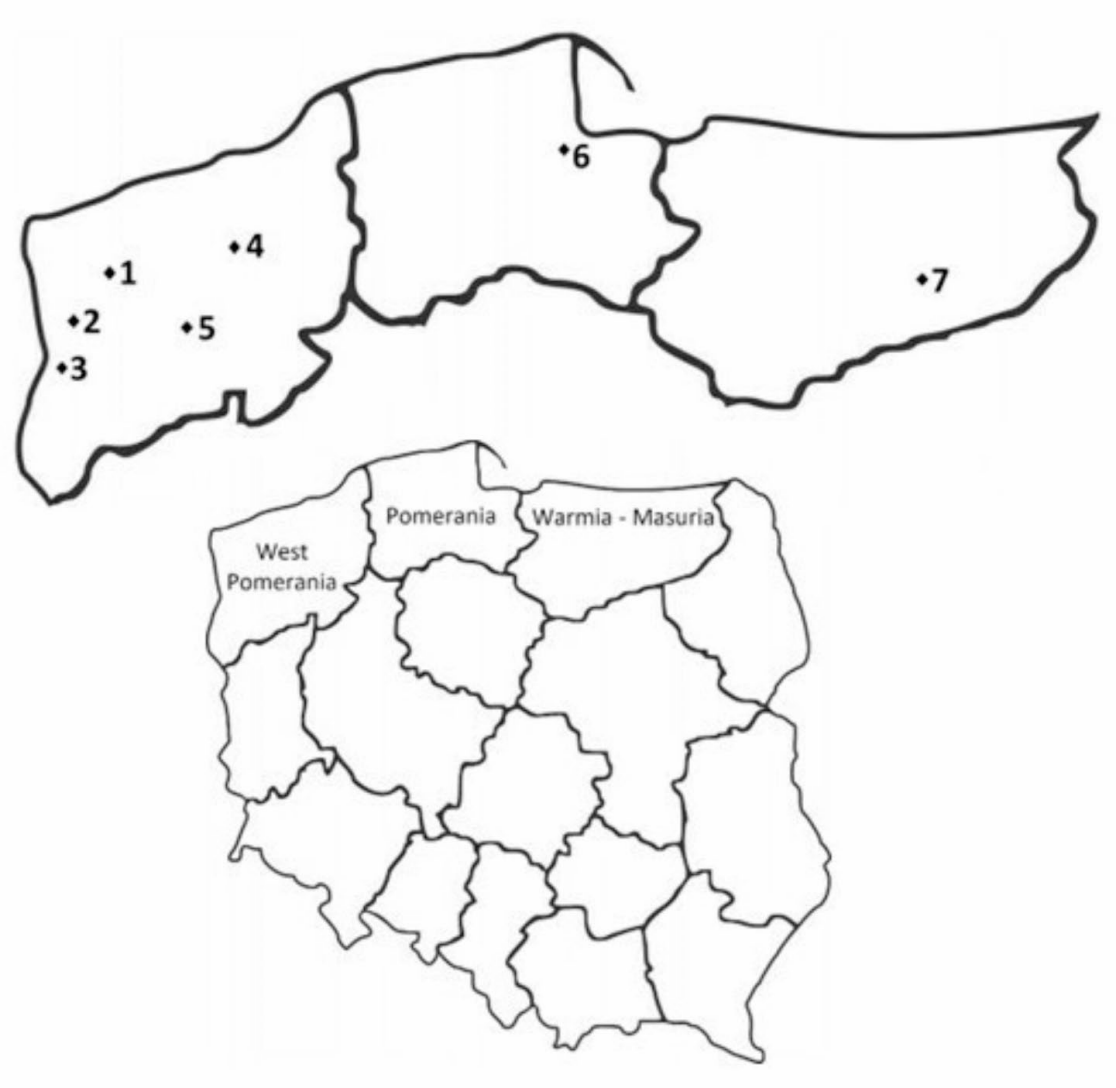
Collection sites of *I. ricinus* ticks. 1 – Zielonczyn (53.6975°N, 14.6661°E), 2 – Bartoszewo (53.5186°N, 14.45722°E), 3 – Lubieszyn (53.4494°N, 14.3892°E), 4 – Świerznica (53.8597°N, 15.9962°E), 5 – Ciemnik (53.3833°N, 15.5667°E), 6 – Gdańsk (54.3520°N, 18.6466°E), 7 – Bełdany Lake (53.7309°N, 21.55462°E)

Questing ticks were collected from vegetation in northern Poland in May 2016 (sites 1, 2, and 7, Fig. 1) and May 2017 (sites 3–6, Fig. 1) using the flagging method. All seven collection sites are located inside mixed forest complexes, in the close vicinity of villages (sites 1–6) or a lake (site 7), within three voivodeships: West Pomerania, Pomerania, and Warmia-Masuria (Fig. 1). The complexes are inhabited by natural tick hosts: wild ungulates, carnivores, rodents, and birds. They are also adjacent to pastures for domestic ruminants, which may come into contact with ticks. The collection sites were selected due to their recreational values, which attract tourists and residents, thus increasing the risk of their contact with ticks.

The 2144 collected ticks (adults, nymphs, and larvae) were stored in tubes containing 70% ethanol, at -20 °C, until further analysis. Before homogenization and DNA extraction, each tick was examined under a microscope with 300x magnification (Smart 5MP Pro Digital Microscope, Delta Optical, Poland). Microscopic observation of the collected ticks was conducted for morphological identification of their stage and species, using the taxonomic keys (Nowak-Chmura 2013; Nowak-Chmura and Siuda 2012; Siuda 1993). It was also determined during the microscopic observation, whether the ticks were engorged or unfed. Details on the number and stages of the ticks collected at individual sites are presented in Table 1. The ticks have previously been tested for *Borreliaceae* bacteria; molecular identification of tick species has been subsequently carried out (Wodecka and Kolomiiets 2023).

**Table 1:** Results of ticks collection and their infection by *T. gondii* detected with nested PCR

Collection site	Number of collected and infected ticks									
	Females		Males		Nymphs		Larvae		All stadies	
	total	positive (n/%)	total	positive (n/%)	total	positive (n/%)	total	positive (n/%)	total	positive (n/%)
1. Zielonczyn	2	0/0	3	0/0	260	3/1.1	37	3/8.8	302	6/2.0
2. Bartoszewo	17	0/0	19	0/0	148	8/5.4	1	0/0	185	8/4.3
3. Lubieszyn	7	0/0	14	0/0	228	1/0.4	14	0/0	263	1/0.4
4. Świerznica	14	0/0	15	0/0	252	2/0.8	194	0/0	475	2/0.4
5. Ciemnik	20	0/0	29	0/0	276	2/0.7	184	0/0	509	2/0.4
6. Gdańsk	11	0/0	13	0/0	237	1/0.4	5	0/0	266	1/0.4
7. Bełdany Lake	16	0/0	27	0/0	101	0/0	-	-	144	0/0
All sites	87	0/0	119	0/0	1502	17/1.1	435	3/0.7	2144	20/0.9

### Molecular analysis

DNA isolation from the ticks examined in this study was performed with the phenol-chloroform method (Wodecka and Kolomiiets 2023). Ticks were placed in individual plastic tubes containing 100 ml of PBS buffer and a stainless steel bead (5 mm diameter) and then homogenized through high-speed shaking (50 Hz/5 minutes) with the use of TissueLyser LT (Qiagen, Germany). The lysis step was carried out at 56 °C for 3 h, in the presence of 2X buffer (0.19 M NH4Cl, 0.011 M KHCO3, 0.024 M EDTA; 500 μl per sample), Lysis buffer (0.017 M SDS, 0.01 M TRIS, 0.01 M EDTA; 100 μl per sample) and proteinase K (20 mg/ml; 1 μl per sample). Then, subsequent centrifugations (9000 rpm/10 minutes) with 300 μl of phenol (BioShop, Canada), 400 μl of phenol-chloroform (1:1), and twice with 300 μl of chloroform (POCh, Poland) were performed. DNA was precipitated from the final supernatant using isopropanol (500 μl per sample). The pellet was rinsed with 70% ethanol (250 μl per sample), air-dried, and finally suspended in Tris-EDTA buffer (pH = 8.0). The obtained DNA samples were stored at -70 °C until the next analysis.

Pools were prepared for PCR analysis (five DNA samples, 5 μl of each per pool) and examined for the presence of *T. gondii* DNA. Individual samples from PCR-positive pools were analysed using the same protocol. The B1 gene was used as a marker and nested PCR was performed to detect *T. gondii* DNA in the pools, and then in individual DNA samples. B1 is a 35-fold repeat gene, highly conserved among different strains of *T. gondii* (Mahittikorn et al. 2005). That ensures high sensitivity of PCR and DNA amplification of all *T. gondii* genotypes. The following sets of primers were used for the analysis: outer F1/R1; 944 bp product and inner F2/R2; 688 bp product (Mahittikorn et al. 2005). Each PCR mixture (total volume of 10 μl) for the first and the second reaction of nested PCR contained 3 pM of each primer (Genomed, Poland), 0.3 nM of each deoxynucleotide triphosphate (EurX, Poland), 1 μl of 10X PCR buffer, 25mM MgCl_2_, 0.5 U of Taq polymerase (GeneON, Germany), and 1 μl of DNA template. The nested PCR conditions for the first and the second reaction were 2 min at 94 °C followed by 35 cycles of 94 °C for 30 s, 56 °C for 30 s, and 72 °C for 90 s, and a final extension at 72 °C for 5 min. *T. gondii* DNA for the positive controls was obtained from a culture of *T. gondii* ME 49 strain, thanks to Dr. Jacek Sroka from the National Veterinary Research Institute, Puławy, Poland.

All PCR-positive samples were sequenced at Macrogen (Netherlands) with the primers F2/R2, under the abovementioned conditions. The obtained sequences were initially aligned with each other as well as with homologous sequences published in the GenBank database using BLAST (www.ncbi.nlm.nih.gov) and then using MEGA 11 software (Pennsylvania State University, USA), with ClustalW (Tamura et al. 2021). Sequence analysis aimed to determine the genotype of *T. gondii* strains detected in this study. The whole amplified sequence analysis was performed instead of SNPs analysis based on the PCR-RFLP technique to obtain more accurate genotyping results.

### Statistical analysis

Statistical analyses were performed using a chi-squared test to investigate the differences in *T. gondii* prevalence between different stages of the examined ticks and between ticks collected in various sites. Statistical significance was defined as *p* < 0.05. Statistica 8.0 software (StatSoft Inc., USA) was used for analysis.

## Results

Morphological identification showed that all examined ticks belong to the species *Ixodes ricinus*. Adult ticks accounted for 9.6% (207/2144) of all individuals, nymphs – 70.1% (1502/2144), and larvae – 20.3% (435/2144). *T. gondii* DNA was detected in 20 of the tested 2144 specimens (0.9%). The infection rate was the highest in the case of nymphs (1.1%; 17/1502) and lower in larvae (0.7%; 3/435). *T. gondii* DNA was not detected in adult ticks. The prevalence of *T. gondii* DNA in ticks collected from individual sites ranged from 0 to 4.3%. Details of tick collection results and their infection by *T. gondii* are presented in Table 1. The sequences obtained from all twenty positive samples were deposited in the GenBank database under accession numbers OR547646 – OR547665.

Statistical analysis did not show significant differences in the prevalence of *T. gondii* DNA between particular stages of the examined ticks (*p*-values > 0.05, Table 2). Ticks collected in Bartoszewo were infected with *T. gondii* significantly more often than those collected from other sites (*p*-values < 0.05, Table 3). Differences between the prevalence of *T. gondii* DNA in ticks collected in Zielonczyn, Lubieszyn, Świerznica, Ciemnik, Gdańsk, and near Bełdany Lake were not statistically significant (*p*-values > 0.05, Table 3).

**Table 2:** *P*-values for *T. gondii* prevalence in different stages of *I. ricinus* ticks

	Females	Males	Nymphs	Larvae
Females	-			
Males	1.0000	-		
Nymphs	0.3262	0.2525	-	
Larvae	0.4433	0.3708	0.4280	-

**Table 3:** *P*-values for *T. gondii* prevalence in *I. ricinus* ticks from different collection sites

	Zielonczyn	Bartoszewo	Lubieszyn	Świerznica	Ciemnik	Gdańsk	Bełdany Lake
Zielonczyn	-						
Bartoszewo	0.1457	-					
Lubieszyn	0.0985	0.0263	-				
Świerznica	0.0516	0.0291	0.9341	-			
Ciemnik	0.0538	0.0302	0.9788	0.9487	-		
Gdańsk	0.0961	0.0262	0.9937	0.9295	0.9714	-	
Bełdany Lake	0.1019	0.0313	0.4646	0.4425	0.4571	0.4671	-

Analysis of the sequences obtained in this study revealed the presence of four polymorphic sites. Their presence allowed the detection of eight variants of the analyzed B1 fragment (Table 4). The substitutions from positions 273, 301, and 394 were found in the intron. The substitution from position 542 was found in the mRNA coding sequence. The latter one turned out to be synonymous. Two sequences available in the Gene Bank database (AF179871, VEG strain, and LN714499) have a sufficient length to overlap with the sequences obtained in this study. The similarity of the sequences obtained in this study and the sequences from the GenBank database ranged between 99.42% and 100% (Table 4).

**Table 4:** Polymorphic sites within the B1 gene sequences described in this study (OR547646 – OR547665)

Sequence variant	Accession number(s)	Polymorphic sites within the sequences OR547646 – OR547665				Identity to the AF179871 sequence	Identity to the LN714499 sequence
		273	301	394	542		
1	OR547646, OR547647	C	C	G	T	99.71%	99.56%
2	OR547648 - OR547651	C	T	G	T	99.56%	99.42%
3	OR547652	C	T	G	C	99.71%	99.56%
4	OR547653	C	T	A	C	99.71%	99.56%
5	OR547654 - OR547658	A	C	A	C	99.85%	100%
6	OR547659 - OR547662	A	C	G	C	100%	99.85%
7	OR547663, OR547664	A	T	G	C	99.85%	99.71%
8	OR547665	A	T	A	C	99.71%	99.85%

Among all twenty ticks positive for *T. gondii*, 30% (one nymph from Świerznica; accession number OR547663, and five nymphs from Bartoszewo; accession numbers OR547650, OR547653, OR547656, OR547658, and OR547662) were positive for bacteria of the *Borreliella* genus (formerly *Borrelia*) that have previously been detected by Wodecka and Kolomiiets (2023). The nymph from Świerznica was coinfected with *Borreliella myiamotoi*, two nymphs from Bartoszewo - *B. carolinensis*, and the remaining three - with *B. garinii*, *B. afzelii*, and *B. spielmanii*. Five B1 variants of *T. gondii* were detected in the six coinfected ticks. The percentage of coinfections with *T. gondii* and *Borreliella* spp. among all examined ticks was 0.3% (6/2144) vs. 0.6% (14/2144) of single *T. gondii* infections.

## Discussion

*I. ricinus* is the most widespread tick species in Europe and one of the arthropods of the most tremendous significance in the epidemiology of transmissible diseases (Nowak-Chmura 2013; Nowak-Chmura and Siuda 2012). The few studies on *T. gondii* presence in *I. ricinus* ticks include fewer specimens collected from a much narrower area than this one. In this study, *T. gondii* prevalence varied depending on the collection site. It was much higher among the ticks collected in Bartoszewo than those collected in other places, even closely situated ones. Furthermore, *T. gondii* was absent in ticks from one collection site. Other studies also revealed significant differences between the infection rate of ticks collected from individual locations, and the lack of *T. gondii* DNA in ticks from some collection sites (Asman et al. 2015, 2017; Sroka et al. 2003, 2008, 2009). These results may indicate a significant impact of local climatic conditions on the occurrence of *T. gondii* in ticks. A similar influence of climatic conditions has been described for the tick bacteriome (Thapa et al. 2018; Tóth et al. 2023).

The overall prevalence of *T. gondii* DNA in questing *I. ricinus* revealed in this study was lower compared to the prevalence in other examined questing populations of this tick species, collected in different parts of Poland (Adamska and Skotarczak 2017; Asman et al. 2015, 2017; Sroka et al. 2003, 2008, 2009). However, Cronhjort et al. (2019) did not detect *T. gondii* DNA in any of the 1849 engorged *I. ricinus* ticks collected from humans in Sweden and Finland, despite the *T. gondii* presence in blood donors in Sweden. The authors conclude that the possible reason may be the very focal occurrence of *T. gondii*. The low percentage of *T. gondii*-positive ticks examined in this study may also be connected with the irregular distribution of this parasite and with fluctuations in the infection level over the years. There are no studies on the influence of environmental conditions on *T. gondii* prevalence in ticks. However, the impact of environmental temperature on the tick bacterial microbiome has been confirmed experimentally (Thapa et al. 2018). Additionally, a relationship was noted between the composition of vector-borne bacteria within *I. ricinus* specimens and climatic conditions at their collection points (Tóth et al. 2023). It is possible that various external factors, such as temperature or humidity, influence *T. gondii* occurrence in ticks. New studies are necessary to determine if external conditions can influence the persistence of *T. gondii* in ticks. There is also a need to investigate whether the prevalence of *T. gondii* in ticks is subject to seasonal and annual fluctuations.

In this study, the infection rate was the highest in nymphs. However, the differences between nymphs, adults, and larvae were not statistically significant. In contrast, other studies including adults and nymphs demonstrate the highest infection rate in females and the lowest in nymphs (Asman et al. 2015; Sroka et al. 2003, 2008, 2009). The lack of *T. gondii* in the examined adult *I. ricinus* may be caused by their small percentage share in the total pool compared to the studies cited above and the low infection rate of all collected ticks. Nymphs may respond differently to environmental conditions (e.g. temperature, humidity, or insolation) than adults and larvae (Wongnak et al. 2022). Distinct conditions during tick collection from various sites may be a reason for different patterns of their questing activity. This may explain the predominance of nymphs among the ticks collected in this study compared to the others.

There are different opinions on the role of ticks in *T. gondii* life cycle. According to some authors, the commonly known transmission routes of *T. gondii* do not fully explain its widespread occurrence in various hosts, especially herbivores. They consider an alternative route of infection via tick bite, as ticks can feed on all warm-blooded animals (Ben-Harari 2019). Zhou et al. (2016) conclude that transmission through infected tick ingestion may explain the common occurrence of *T. gondii* in different hosts. According to them, toxoplasmosis may not be a tick-borne disease, but the possibility of transmission by infected larvae and/or nymphs cannot be ruled out. Experimental studies proving tick-borne transmission of *T. gondii* are very few (Deryło et al. 1978; Sroka 2008, Woke et al. 1953). *T. gondii* proliferation in some tissues of females (i.a. salivary glands) and nymphs of *I. ricinus* was shown (Deryło et al. 1978). Nevertheless, several authors do not accept the possibility of *T. gondii* propagation in the tissues of poikilothermic animals (Dubey and Beattie 1988; Jira and Rosický 1983). The presence of *T. gondii* in the ticks examined in this study does not indicate that they are competent vectors for the parasite. However, they could pose a source of infection for mammals and birds that can eat ectoparasites residing in the environment or feeding on the hosts (Gryczyńska et al. 2024; Zhou et al. 2016). This may be an additional, minor part of the *T. gondii* transmission cycle. Finding the presence of *T. gondii* in unfed larvae and nymphs revealed in this study is significant. It may indicate the possibility of its vertical (transovarial and transstadial) transmission, which was suggested earlier (Ben-Harari 2019; Gryczyńska et al. 2024). Experimental studies are needed to establish whether *T. gondii* transmission by blood-feeding infected ticks is possible, or whether its presence in a tick’s body is a dead end for the parasite.

The ticks examined in this study have been tested previously to detect the presence of *Borreliaceae* bacteria DNA, as representatives of this family are the most frequent species found in ticks. They are causative agents of Lyme borreliosis, one of the most common tick-borne diseases within the Northern Hemisphere, and relapsing fever (Wodecka and Kolomiiets 2023). The overall percentage of coinfected ticks was low, but as many as one-third of all ticks infected with *T. gondii* were also infected with *Borreliella* spp. *T. gondii* can infect any warm-blooded animal and occurs in various intermediate hosts (Ben-Harari 2019; Warschkau and Seeber 2023), so it shares some hosts with *Borreliella* bacteria. The main European reservoir of *Borreliella* species that coinfected the examined ticks with *T. gondii* are mainly rodents and, to a lesser extent, insectivores and birds (Cleveland et al. 2023; Steinbrink et al. 2022). These vertebrates may also be a source of *T. gondii* infection for *I. ricinus* ticks from the examined area. Their involvement in *T. gondii* maintenance in the environment was suggested by Gryczyńska et al. (2024) and Sroka et al. (2019). Comprehensive studies on *T. gondii* occurrence in particular species of vertebrates, ticks infesting them, and questing ticks from the area where they occur would contribute to a better understanding of the role of different intermediate host species in the *T. gondii* life cycle.

The B1 sequences obtained in this study show the greatest similarity to the sequence derived from the VEG strain of *T. gondii*, representing type III (Quiarim et al. 2021). Type II predominates in Europe followed by type III, concerning all types of the samples examined so far. On the other hand, type I predominates in European ticks, regardless of the marker used for genotyping (Fernández-Escobar et al. 2022). The studies on *T. gondii* genotyping in European ticks are very few (Adamska and Skotarczak 2017; Sroka et al. 2008, 2009; Wójcik-Fatla et al. 2015) and they may not reflect the full genetic diversity of *T. gondii* occurring in these arthropods. Genotyping of *T. gondii* from more samples is necessary to discover the real genetic structure of its populations in European ticks. In this study, type III was detected in the examined samples. Thus, the genetic diversity of the *T. gondii* population in European ticks may be higher than so far described. However, the widely used methods for *T. gondii* genotyping, including multilocus sequence analysis, may give ambiguous results. Unsuccessful amplification for many markers used for the multilocus analysis also occurs due to their low sensitivity (Battisti et al. 2018; Fernández-Escobar et al. 2022; Sroka et al. 2017, 2019). Thus, the results of *T. gondii* genotyping should be cautiously assumed, regardless of the markers analysed. According to Fernández-Escobar et al. (2022), whole-genome sequencing (WGS) data analysis would be the most suitable tool for the genetic analysis of *T. gondii*. They also conclude that more WGS data are needed, as they are available only for a few European isolates. However, high WGS costs are still the reason for the low amount of WGS data and significantly hinder detailed research on *T. gondii* genetic diversity.

## Conclusions

Questing *I. ricinus* ticks from northern Poland harbour *T. gondii* and after ingestion may pose a source of infection for vertebrates. Small vertebrates, such as rodents, insectivores, and birds, should be considered a source of *T. gondii* infection for *I. ricinus* ticks. The prevalence of *T. gondii* in *I. ricinus* ticks is focal and may fluctuate over time. There is a possibility of vertical transmission of the parasite. The detected strains of *T. gondii* are the most similar or identical to type III, and the genetic diversity of *T. gondii* in European ticks may be higher than so far described.

## Data Availability

Sequence data that support the findings of this study have been deposited in GenBank database with the accession numbers OR547646 - OR547665.

## References

[CR1] Adamska M, Skotarczak B (2017) Molecular evidence for *Toxoplasma Gondii* in feeding and questing *Ixodes ricinus* ticks. Ticks Tick Borne Dis 8:259–261. 10.1016/j.ttbdis.2016.11.009 27894863 10.1016/j.ttbdis.2016.11.009

[CR2] Asman M, Solarz K, Cuber P, Gąsior T, Szilman P, Szilman E, Tondaś E, Matzullok A, Kusion N, Florek K (2015) Detection of protozoans *Babesia microti* and *Toxoplasma Gondii* and their coexistence in ticks (Acari: Ixodida) collected in Tarnogórski district (Upper Silesia, Poland). Ann Agric Environ Med 22:80–83. 10.5604/12321966.1141373 25780833 10.5604/12321966.1141373

[CR3] Asman M, Nowak-Chmura M, Solarz K, Szilman E, Semla M, Zyśk B (2017) Anaplasma phagocytophilum, Babesia microti, Borrelia burgdorferi Sensu Lato, and Toxoplasma Gondii in Ixodes ricinus (Acari, Ixodida) ticks collected from Slowinski National Park (Northern Poland). J Vector Ecol 42:200–202. 10.1111/jvec.12258 28504439 10.1111/jvec.12258

[CR4] Battisti E, Zanet S, Trisciuoglio A, Bruno S, Ferroglio E (2018) Circulating genotypes of *Toxoplasma Gondii* in Northwestern Italy. Vet Parasitol 253:43–47. 10.1016/j.vetpar.2018.02.023 29605002 10.1016/j.vetpar.2018.02.023

[CR5] Ben-Harari RR (2019) Ticks transmission of toxoplasmosis. Expert Rev Anti Infect Ther 17:911–917. 10.1080/14787210.2019.1682550 31623503 10.1080/14787210.2019.1682550

[CR6] Cleveland DW, Anderson CC, Brissette CA (2023) *Borrelia miyamatoi*: a comprehensive review. Pathogens 12:267. 10.3390/pathogens12020267 36839539 10.3390/pathogens12020267 PMC9967256

[CR7] Cronhjort S, Wilhelmsson P, Karlsson L, Thelaus J, Sjӧdin A, Forsberg P, Lindgren P-E (2019) The tick-borne diseases STING study: real-time PCR analysis of three emerging tick-borne pathogens in ticks that have bitten humans in different regions of Sweden and the Aland Islands, Finland. Infect Ecol Epidemiol 9:1683935. 10.1080/20008686.2019.1683935 31741721 10.1080/20008686.2019.1683935 PMC6844441

[CR8] Deryło A, Toś-Luty S, Dutkiewicz J, Umiński J (1978) Researches in participation of ticks *Ixodes ricinus* L. in biology and transmission of *Toxoplasma Gondii*. Wiad Parazytol 24:585–596 741744

[CR10] du Plooy I, Mlangeni M, Christian R, Tsotetsu-Khambule AM (2023) An African perspective on the genetic diversity of *Toxoplasma Gondii*: a systematic review. Parasitology 150:551–578. 10.1017/S0031182023000252 36938833 10.1017/S0031182023000252 PMC10260301

[CR9] Dubey JP, Beattie CP (1988) Toxoplasmosis of animals and men. CRC Press Inc., Boca Raton

[CR11] Ergunay K, Mutinda M, Bourke B, Justi SA, Caicedo-Quiroga L, Kamau J, Mutura S, Akunda IK, Cook E, Gakuya F, Omondi P, Murray S, Zimmerman D, Linton Y-M (2022) Metagenomic investigation of ticks from Kenyan wildlife reveals diverse microbial pathogens and new country pathogen records. Front Microbiol 13:932224. 10.3389/fmicb.2022.932224 35847110 10.3389/fmicb.2022.932224 PMC9283121

[CR12] Fernández-Escobar M, Schares G, Maksimov P, Joeres M, Ortega-Mora LM, Calero-Bernal R (2022) *Toxoplasma gondii* genotyping: a closer look into Europe. Front Cell Infect Microbiol 12:842595. 10.3389/fcimb.2022.842595 35402301 10.3389/fcimb.2022.842595 PMC8984497

[CR13] Gryczyńska A, Polaczyk J, Welc-Falęciak R (2024) *Toxoplasma gondii* infection in ticks infesting migratory birds: the blackbird (*Turdus merula*) and the song thrush (*Turdus philomelos*). Exp Appl Acarol: Published Online. 10.1007/s10493-023-00878-0 10.1007/s10493-023-00878-0 38321308

[CR14] Jagow M, Hoffmann G (1970) Untersuchungen Zur Übertragung Von Toxoplasma Gondii Durch Verschiedene Entwicklungsstadienvon Ornothodoros moubata. Z Parasitenkde 33:246–251 10.1007/BF00259494 5426824

[CR15] Jira J, Rosický B (1983) Immunodiagnostika a epidemiologic toxoplasmosy. Academia, Prague. (in Czech)

[CR16] Kim JY, Kwak YS, Lee I-Y, Yong T-S (2020) Molecular detection of *Toxoplasma Gondii* in *Haemaphysalis* ticks in Korea. Korean J Parasitol 58:327–331. 10.3347/kjp.2020.58.3.327 32615747 10.3347/kjp.2020.58.3.327 PMC7338900

[CR17] Kocoń A, Asman M, Nowak-Chmura M, Witecka J, Kłyś M, Solarz K (2020) Molecular detection of tick-borne pathogens in ticks collected from pets in selected mountainous areas of Tatra County (Tatra Mountains, Poland). Sci Rep 10:15865. 10.1038/s41598-020-72981-w 32985586 10.1038/s41598-020-72981-w PMC7522974

[CR18] Mahittikorn A, Wickert H, Sukthana Y (2005) Comparison of five DNA extraction methods and optimization of a B1 gene nested PCR (nPCR) for detection of *Toxoplasma gondii* tissue cyst in mouse brain. Southeast Asian J Trop Med Public Health 36:1377–1382 16610637

[CR19] Nowak-Chmura M (2013) The Fauna of Ticks (*Ixodida*) of Central Europe. Wydawnictwo Naukowe Uniwersytetu Pedagogicznego, Kraków

[CR20] Nowak-Chmura M, Siuda K (2012) Ticks of Poland. Review of contemporary issues and latest research. Ann Parasitol 58:125–155 23444797

[CR21] Quiarim TM, Maia MM, da Cruz AB, Taniwaki NN, Namiyama GM, Pereira-Chioccola VL (2021) Characterization of extracellular vesicles isolated from types I, II and III strains of *Toxoplasma Gondii*. Acta Trop 219:105915. 10.1016/j.actatropica.2021.105915 33861971 10.1016/j.actatropica.2021.105915

[CR22] Siuda K (1993) Ticks (Acari: Ixodida) of Poland. Part II: taxonomy and distribution. Polskie Towarzystwo Parazytologiczne, Warsaw

[CR23] Sroka J, Chmielewska-Badora J, Dutkiewicz J (2003) *Ixodes ricinus* as a potential vector of *Toxoplasma gondii*. Ann Agric Environ Med 10:121–123 12852744

[CR24] Sroka J, Wójcik-Fatla A, Zwoliński J, Zając V, Sawczuk M, Dutkiewicz J (2008) Preliminary study on the occurrence of *Toxoplasma Gondii* in *Ixodes ricinus* ticks from North-Western Poland with the use of PCR. Ann Agric Environ Med 15:333–338 19061272

[CR25] Sroka J, Szymańska J, Wójcik-Fatla A (2009) The occurrence of *Toxoplasma Gondii* and *Borrelia burgdorferi* Sensu Lato in *Ixodes ricinus* ticks from Eastern Poland with the use of PCR. Ann Agric Environ Med 16:313–319 20047269

[CR26] Sroka J, Kusyk P, Bilska-Zając E, Karamon J, Dutkiewicz J, Wójcik-Fatla A, Zając V, Stojecki K, Różycki M, Cencek T (2017) Seroprevalence of *Toxoplasma gondii* infection in goats from the south-west region of Poland and the detection of *T. Gondii* DNA in goat milk. Folia Parasitol 64:023. 10.14411/fp.2017.023 10.14411/fp.2017.023 28783032

[CR27] Sroka J, Karamon J, Wójcik-Fatla A, Dutkiewicz J, Bilska-Zając E, Zając V, Piotrowska W, Cencek T (2019) *Toxoplasma gondii* infection in selected species of free-living animals in Poland. Ann Agric Environ Med 26:656–660. 10.26444/aaem/114930 31885241 10.26444/aaem/114930

[CR28] Steinbrink A, Brugger K, Margos G, Kraiczy P, Klimpel S (2022) The evolving story of *Borrelia burgdorferi* Sensu Lato transmission in Europe. Parasitol Res 121:781–803. 10.1007/s00436-022-07445-3 35122516 10.1007/s00436-022-07445-3 PMC8816687

[CR29] Tamura K, Stecher G, Kumar S (2021) MEGA11: molecular evolutionary genetics analysis version 11. Mol Biol Evol 38:3022–3027. 10.1093/molbev/msab120 33892491 10.1093/molbev/msab120 PMC8233496

[CR30] Thapa S, Zhang Y, Allen MS (2018) Effects on temperature on bacterial microbiome composition in *Ixodes scapularis* ticks. Microbiologyopen 8:e719. 10.1002/mbo3.719 10.1002/mbo3.719 PMC6528569 30239169

[CR31] Tóth AG, Farkas R, Papp M, Kilim O, Yun H, Makrai L, Maróti G, Gyurkovszky M, Krikó E, Solymosi N (2023) Ixodes ricinus tick bacteriome alterations based on a climatically representative survey in Hungary. Microbiol Spectr 11:1–17. 10.1128/spectrum.01243-23 10.1128/spectrum.01243-23 PMC10715062 37966205

[CR32] Truong A-T, Yoo M-S, Min S, Lim J-Y, Seo H-J, Kim H-C, Chong S-T, Klein TA, Park C-U, Cho S-Y, Choi C-Y, Kwon Y-S, Kim M, Yoon S-S, Cho YS (2022) *Toxoplasma gondii* and *Rickettsia* spp. in ticks collected from migratory birds in the Republic of Korea. Sci Rep. 12:12672. 10.1038/s41598-022-16785-0 10.1038/s41598-022-16785-0 PMC9314388 35879387

[CR33] Warschkau D, Seeber F (2023) Advances towards the complete in vitro life cycle of *Toxoplasma Gondii*. Fac Rev 12(1). 10.12703/r/12-1 10.12703/r/12-1 PMC9944905 36846606

[CR34] Wodecka B, Kolomiiets V (2023) Genetic diversity of *Borreliaceae* species detected in natural populations of *Ixodes ricinus* ticks. North Pol Life 13:972. 10.3390/life13040972 10.3390/life13040972 PMC10143352 37109501

[CR37] Wójcik-Fatla A, Sroka J, Zając V, Sawczyn A, Cisak E, Dutkiewicz J (2015) *Toxoplasma gondii* (NiEtlle et Manceaux, 1908) detected in *Dermacentor reticulatus* (Fabricius) (Ixodidae). Folia Parasitol 62:005. 10.14411/fp.2015.055 10.14411/fp.2015.055 26449345

[CR35] Woke PA, Jacobs L, Jones FE, Melton ML (1953) Experimental results on possible arthropod transmission of Toxoplasmosis. J Parasitol 39(5):523–532 13097287

[CR36] Wongnak P, Bord S, Jacquot M, Agoulon A, Beugnet F, Bournez L, Cèbe N, Chevalier A, Cosson J-F, Dambrine N, Hoch T, Huard F, Korboulewsky N, Lebert I, Madouasse A, Mårell A, Moutailler S, Plantard O, Pollet T, Poux V, René-Martellet M, Vayssier-Taussat M, Verheyden H, Vourch G, Chalvet-Monfray K (2022) Meteorological and climatic variables predict the phenology of *Ixodes ricinus* nymph activity in France, accounting for habitat heterogeneity. Sci Rep 12:7833. 10.1038/s41598-022-11479z 35552424 10.1038/s41598-022-11479-z PMC9098447

[CR38] Zając V, Wójcik-Fatla A, Sawczyn A, Cisak E, Sroka J, Kloc A, Zając Z, Buczek A, Dutkiewicz J, Bartosik K (2017) Prevalence of infections and co-infections with 6 pathogens in *Dermacentor reticulatus* ticks collected in eastern Poland. Ann Agric Environ Med 24:26–32. 10.5604/12321966.1233893 28378977 10.5604/12321966.1233893

[CR39] Zhou Y, Zhang H, Cao J, Gong H, Zhou J (2016) Epidemiology of toxoplasmosis: role of the tick *Haemaphysalis longicornis*. Infect Dis Poverty 5:14. 10.1186/s.40249-016-0106-0 26897021 10.1186/s40249-016-0106-0 PMC4761159

